# 3D printed composite model of pelvic osteochondroma and nerve roots

**DOI:** 10.1186/s41205-021-00121-9

**Published:** 2021-09-25

**Authors:** Olivia Fox, Andrew Kanawati

**Affiliations:** 1 Department of Orthopaedics, Westmead Hospital, Hawkesbury Road, Westmead, NSW 2145 Australia; 2 Harbour Spine Surgeons, 207 Pacific Highway, St Leonards, NSW 2065 Australia

**Keywords:** Composite, Pelvic tumour, Model, 3D, 3D-printing, Spine, Nerve

## Abstract

**Background:**

3D-printing has become increasingly utilized in the preoperative planning of clinical orthopaedics. Surgical treatment of bone tumours within the pelvis is challenging due to the complex 3D bone structure geometry, as well as the proximity of vital structures. We present a unique case where a composite bone and nerve model of the lower lumbar spine, pelvis and accompanying nerve roots was created using 3D-printing. The 3D-printed model created an accurate reconstruction of the pelvic tumour and traversing nerves for preoperative planning and allowed for efficient and safe surgery.

**Case presentation:**

We present a unique case where a composite bone and nerve model of the lower lumbar spine, pelvis and accompanying nerve roots was created using 3D-printing. The bony pelvis and spine model was created using the CT, whereas the nerve roots were derived from the MRI and printed in an elastic material. 3D-printed model created an accurate reconstruction of the pelvic tumour and traversing nerves for preoperative planning and allowed for efficient and safe surgery. Pelvic tumour surgery is inherently dangerous due to the delicate nature of the surrounding anatomy. The composite model enabled the surgeon to very carefully navigate the anatomy with a focused resection and extreme care knowing the exact proximity of the L3 and L4 nerve roots.

**Conclusion:**

The patient had complete resection of this tumour, no neurological complication and full resolution of his symptoms due to careful, preoperative planning with the use of the composite 3D model.

## Background

3D printing has become increasingly utilized in the preoperative planning of clinical orthopaedics, orthopaedic trauma and other disciplines over the past decade [2]. Surgical treatment of bone tumours within the pelvis is challenging due to the complex 3D bone structure geometry, as well as the proximity of vital structures such as blood vessels, nerve roots, sciatic and femoral nerves and the bladder and/or rectum.

Reproducing the pre-operative plan as accurately as possible is crucial in pelvic tumour surgery, in order to achieve negative surgical margins and thus decrease the likelihood of local recurrence, and reduce the risk of damage to vital structures [1, 12, 15, 18]. However, resecting significantly more tissue than planned, out of concern for leaving a positive margin, can compromise patient function and/or successful reconstruction [12]. Thus, accuracy in executing the pre-operative plan is crucial for safe surgical margins, preserving maximum bone stock, reducing surgical morbidity by allowing approach planning and increase understanding of nearby vital structures.

**Fig. 1:**
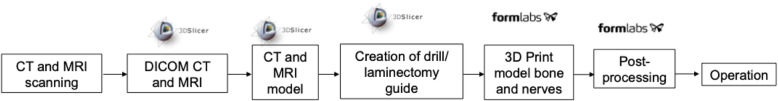
Flow sheet demonstrating the creation and use of the composite model

We present a unique case where a composite bone and nerve model of the lower lumbar spine, pelvis and accompanying nerve roots were segmented using 3D Slicer version 4.10.2 (www.slicer.org), and 3D-printed using a Formlabs Form 2 (Formlabs Inc. Somerville, MA) desktop 3D printer. The bony pelvis and spine were segmented using computed tomography (CT) data and printed in a solid material, whereas the nerve roots were segmented from magnetic resonance imaging (MRI) data, and printed in an elastic material (Fig. 1). The 3D-printed model created an accurate reconstruction of the pelvic tumour and traversing nerves for preoperative planning and allowed for efficient and safe surgery.

## Case presentation

A 40 year-old male presented to our institution with a 20-year history of mild left-sided lower back and flank pain, secondary to an osteochondroma. His pain was activity related and was gradually increasing over 2 years. He occasionally complained of nocturnal symptoms.

**Fig. 2:**
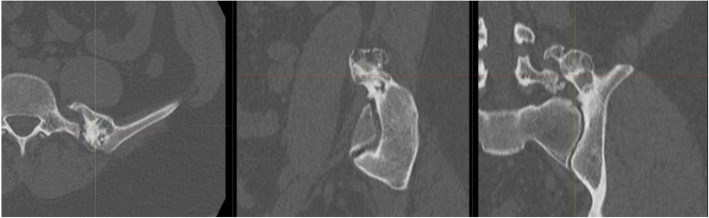
CT slices demonstrating the bony architecture of the osteochondroma

**Fig. 3:**
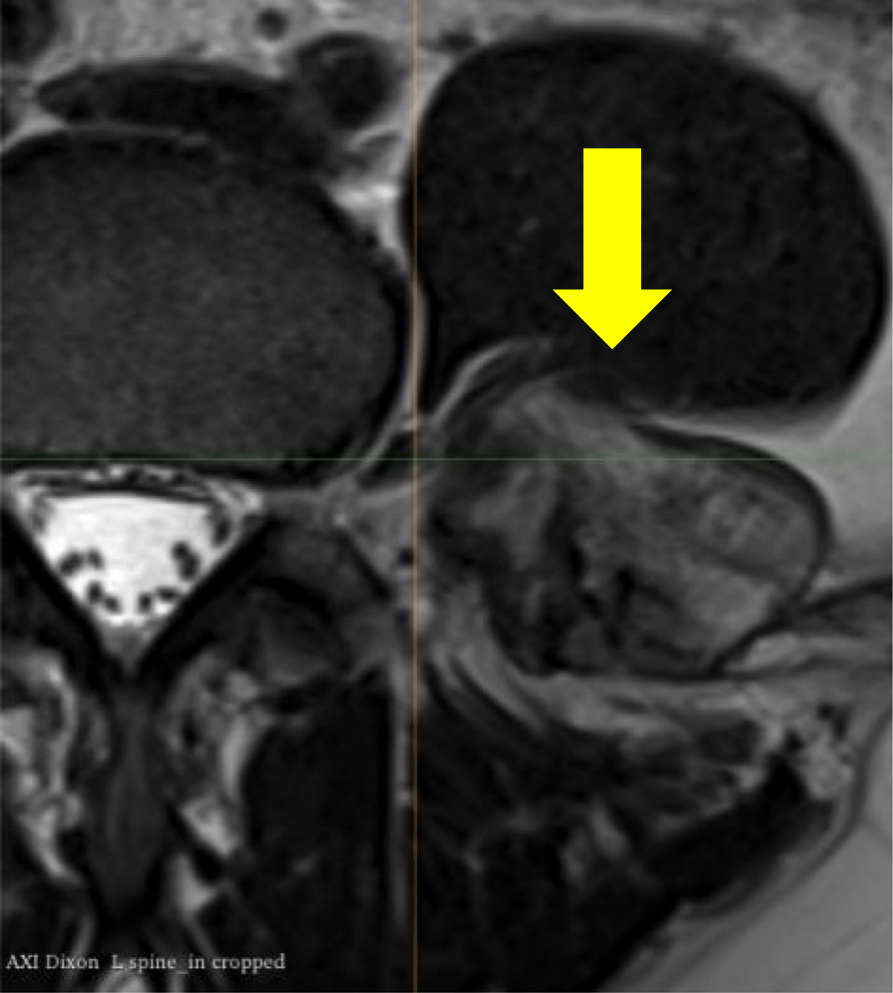
MRI demonstrating the L3 nerve root anterior to the osteochondroma (arrow)

Radiological imaging included serial CT and MRI of the lumbar spine (Fig. 2 and 3). The CT data was obtained using a GE Lightspeed VCT 64 slice CT Scanner with 0.625 mm slice thickness. The MRI data was obtained using a GE Healthcare 3 T Discovery MR750 scanner. The scan parameters for each modality are outlined in Tables 1 and 2. The most recent scans revealed a lesion originating from the posteromedial aspect of the left iliac crest lesion, adjacent to and surrounding the left L5 transverse process. It had a stable appearance since the patient’s puberty and had no aggressive radiological features of chondrosarcoma. The MRI revealed the proximity of the L3, L4 and L5 nerve roots as they exited the intervertebral foraminae and before they passed into the psoas muscle.

**Table 1:** MRI imaging parameters

Imaging options	Parameters
Imaging Plane	Oblique
Imaging Mode	3D
Pulse Sequence	Cube
Imaging Options	EDR, Fast, ZIP2, FR
Frequency Field of View	300 mm
Phase Field of View	60% or 300 mm
Frequency Matrix	384 mm
Phase Matrix	384 mm
Slice Thickness	0.8 mm
Slice Overlap	None
NEX	2
Flip Angle	5 Degrees
TR	4800 ms
TE	369 ms
Receive Bandwidth	50 kHz
Frequency Direction	S/I

**Table 2:** CT imaging

Imaging options	Parameters
Pitch	0.984:1
Table speed	39.37 mm/ro
Helical Full	1.0 s
SFOV	Large Body
DFOV	40 adjust as needed
Auto mA	ON
Smart mA	ON
Min mA	200
Max mA	650
Dose Reduction	20%
Noise Index	26
ASIR	40%

The patient was conservatively managed however after 5 months he represented with progression of his symptoms reporting depressive symptoms and cessation of work because of the pain. He started to complain of pain and numbness in his thigh and knee, due to L3 and L4 radiculopathies. He responded well to selective nerve root injections, however his symptoms recurred and operative management was decided upon.

**Fig. 4:**
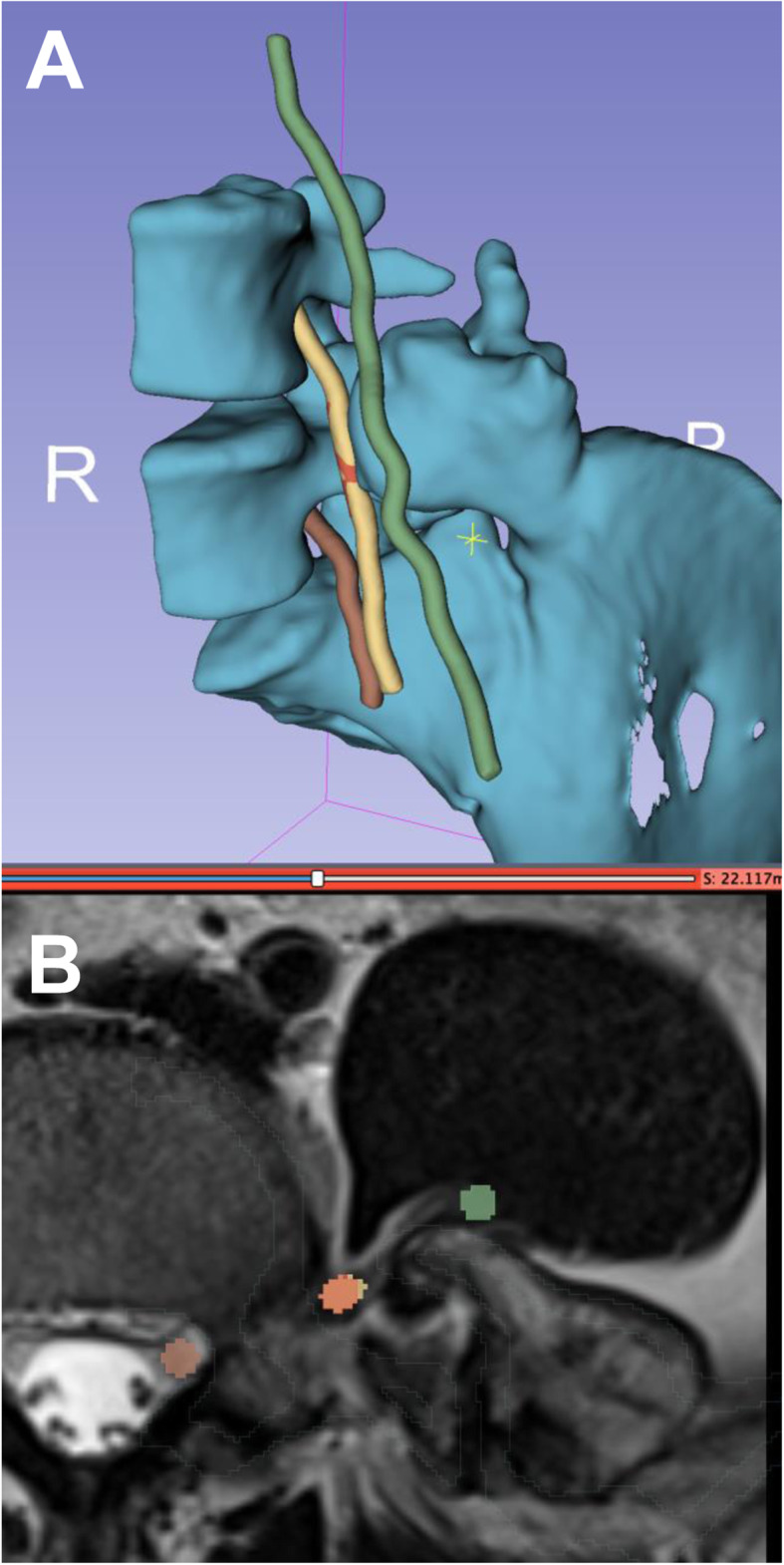
**A** Composite segmentation model of the left ilium osteochondroma based on CT data, and L3–5 nerve roots based on MRI data (**B**)

**Fig. 5:**
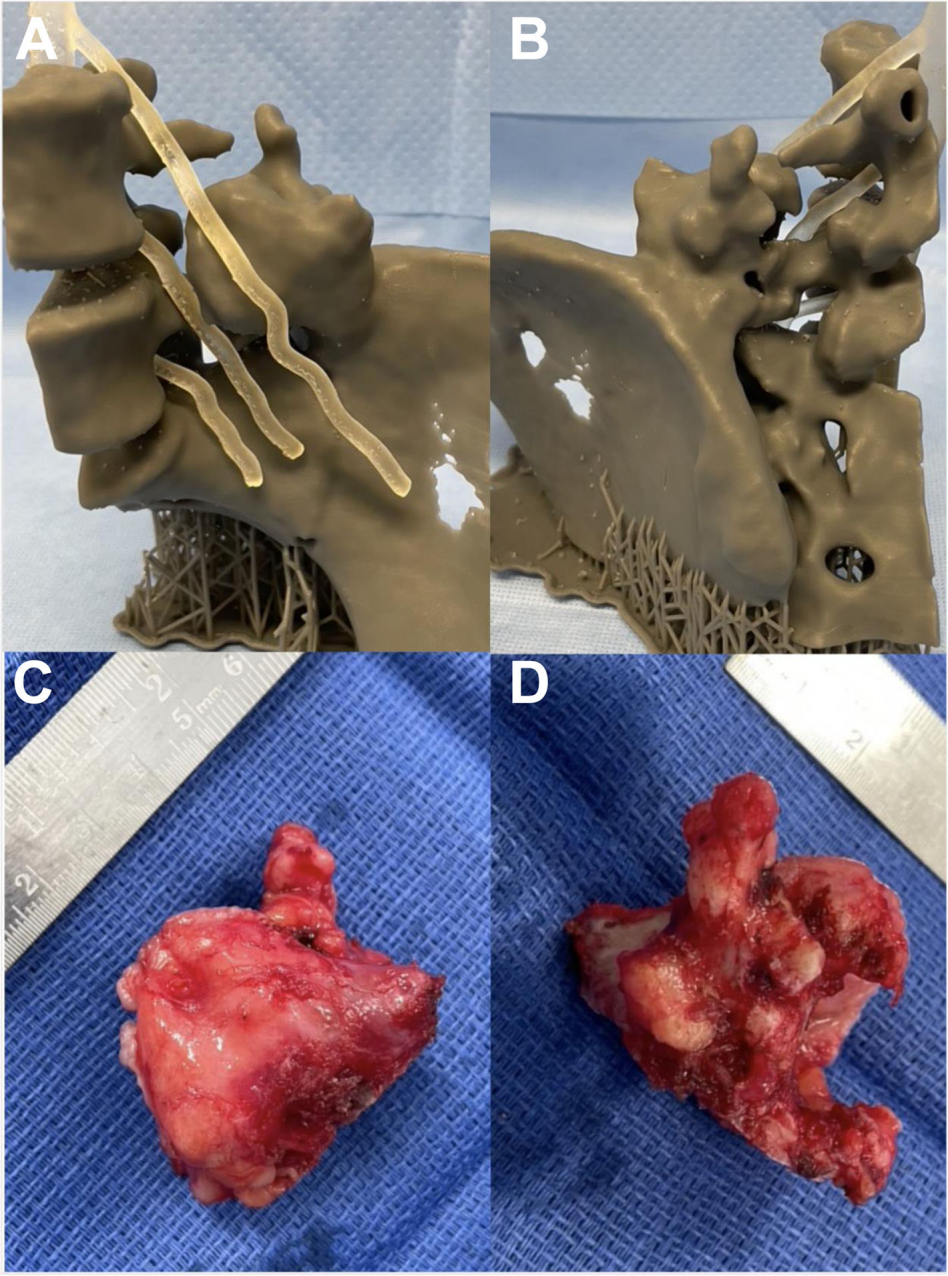
3D-printed model combining the CT and the MRI elements demonstrating the exact proximity of the nerve roots to the tumour. Anterior (**A**) and posterior (**B**) views. Corresponding anterior (**C**) and posterior (**D**) views of the excised osteochondroma

3D-printing of the pelvis CT and lumbar spine MRI were used for preoperative planning (Fig. 4). The CT digital imaging and communication in medicine (DICOM) was imported into 3D Slicer version 4.10.2. A region of interest was created around the left ilium, sacrum, L4 and L5 vertebrae, with a crop scale of 1.0 and isotropic spacing. The model was created by the ‘grow from seeds’ extension in the Segment Editor of 3D Slicer. Segmentation defects were corrected by modifying seeds and manual editing to ensure accuracy of the models. Closing (fill holes) and opening (remove extrusions) smoothing effects at a kernel size of 2 mm were used to obtain a final model. The model was made hollow with a shell thickness of 2 mm, and was exported as a standard tessellation language (STL) file. The MRI DICOM was imported into 3D Slicer. The axial T2 weighted sequences were used to identify the nerve roots. The ‘draw tube’ extension in the Segment Editor of 3D Slicer was used to trace the L3, L4 and L5 nerve roots using a 2 mm radius. The nerve roots were combined into one model and exported as an STL file. The bone and nerve models were aligned to allow for closer assessment of the nerve roots relationship to the tumour. The L3 nerve was found to be traversing directly anterior to the lesion and actually form a groove on the anterior and cranial surface (Figs. 4 and 5). The L4 nerve root was found to be traversing directly medial to the lesion.

The bone model STL file was imported into Formlabs Preform software (Version 3.0.2). Layer thickness was set to 0.1 mm, supports were autogenerated and printed in Grey resin using a Formlabs Form 2 (Formlabs Inc. Somerville, MA) desktop 3D printer. The print time was 13 h and 15 min, and print volume was 176 mls.

The nerve root model STL file was imported into Formlabs Preform software. Layer thickness was set to 0.1 mm, supports were autogenerated and printed in Elastic 50A resin, using a Formlabs Form 2 desktop 3D printer. The print time was 3 h and 45 min, and print volume was 18 mls. After printing, post-processing of the models included soaking in 99% isopropyl alcohol for 20 min, and then UV curing for 30 min at 60-degrees Celsius in the Form Cure machine (Formlabs Inc. Somerville, MA). After removal of supports, the models were inspected to exclude printing failures or errors. No additional postprocessing was required.

The patient was positioned prone on a Jackson table after administration of a general anaesthetic. The tumour was approach via a posterior, longitudinal, paramedian incision (Wiltse approach). The posterior aspect of the tumour was dissected in a subperiosteal manner. A bone scalpel (Misonix, Farmingdale, NY) was used to make one osteotomy at the base of the lesion, adjacent to the superomedial aspect of the ilium. The lesion was removed en-bloc. Complete resection of the lesion was confirmed by comparing the resected lesion to the 3D-printed model. Operative resection of the tumour took 58 mins, and was confirmed an osteochondroma on histology with negative surgical margins (Fig. 5). There were no intra- or postoperative complications, with complete resolution of symptoms at 2 weeks and 3 months postoperatively.

## Discussions and conclusion

This case demonstrates a unique composite spine and pelvis model using a combination of 3D-printing of the exiting nerves from MRI scan data and the bony architecture from CT scan data. The use of elastic material to recreate neural soft tissue is a novel technique, and this elastic material can also be used to mimic other musculoskeletal soft tissue such as ligaments and tendons. Multiple pre-operative benefits included explaining the operative risk of iatrogenic nerve injury to the patient, but also provided a guide for intra-operative management.

Several procedures for improving surgical accuracy have been described, such as computer-assisted surgical navigation, robot-assisted surgery and use of 3D-printed patient-specific guides [5, 9, 12, 19, 20]. Several studies have validated CT and MRI in the creation of accurate 3D-printed models, as well as the use of 3D Slicer in the creation of musculoskeletal segmentation. Several of our previous reports have demonstrated the dimensional and volumetric accuracy of both CT and MRI data when compared to bone and 3D-printed models [10, 11]. In a comparison of the 3D model and the cadaver pelvis, 3D printing resulted in accurate models suitable for preoperative workup [4]. 3D printing contributes to a better understanding of the surgical approach, reduction and fixation of fractures, especially in complex fractures such as acetabular fractures [3, 7, 13, 14, 16]. Furthermore, more accurate reduction and shorter operation times can be achieved [6, 21]. Additionally multiple cadaveric studies on pelvic tumours demonstrated more accurate osteotomies with 3D-printed patient-specific instruments compared to the standard manual technique [8, 17].

Pelvic tumour surgery is inherently dangerous due to the delicate nature of the surrounding anatomy. The composite model enabled the surgeon to very carefully navigate the anatomy with a focused resection and extreme care knowing the exact proximity of the L3 and L4 nerve roots. The patient had complete resection of this tumour, no neurological complication and full resolution of his symptoms due to careful preoperative planning with the use of the composite 3D model.

## Data Availability

Not applicable.
